# Self-Determination Theory and Quality of Life of Adults with Diabetes: A Scoping Review

**DOI:** 10.1155/2023/5341656

**Published:** 2023-04-12

**Authors:** Jacob Owusu Sarfo, Paul Obeng, Henneh Kwaku Kyereh, Edward Wilson Ansah, Priscilla Yeye Adumoah Attafuah

**Affiliations:** ^1^Department of Health, Physical Education and Recreation, University of Cape Coast, Ghana; ^2^Public Health Nursing Department, School of Nursing and Midwifery, University of Ghana, Ghana

## Abstract

**Background:**

Diabetes is one of the leading causes of sickness, death, and decreased quality of life globally. The prevalence of diabetes keeps rising globally due to lifestyle changes and urbanization. Therefore, improved quality of life (QoL) and appropriate diabetes self-management practices, including treatment adherence, are crucial to improving and sustaining the health of diabetic patients. Some studies have adopted the self-determination theory (SDT) to study diabetes interventions, but less is known about its effectiveness in improving QoL, treatment adherence, and diabetes self-management. *Aim/Objective*. This review assessed the effectiveness of SDT in improving self-management practices, treatment adherence, and QoL among adult diabetic patients.

**Method:**

We followed the six-stage framework by Arksey and O'Malley in conducting the review. PubMed, JSTOR, Central, and ScienceDirect databases were searched for published articles from January 2011 to October 2021 using keywords and Boolean logic. Furthermore, we screened a reference list of related articles. Also, Google Scholar, Z-library, and web-based searches were carried out to retrieve other relevant evidence that applied SDT in improving QoL, diabetes self-management, and treatment adherence. *Findings*. Fifteen studies met the inclusion criteria, from which data were extracted as findings. SDT effectively improved QoL, diabetes treatment adherence, and diabetes self-management among diabetic patients. Of these studies, 11 provided data on SDT and diabetes self-management and affirmed the effectiveness of the theory in improving appropriate diabetes self-management practices. Two studies confirmed the effectiveness of SDT in improving treatment adherence. SDT and QoL were assessed in 4 of the studies, which demonstrated the effectiveness of SDT in enhancing the QoL of diabetic patients.

**Conclusion:**

SDT effectively improved QoL, diabetes treatment adherence, and diabetes self-management. The application of SDT in diabetes management will improve the health and QoL of diabetic patients. Hence, diabetes management interventions could adopt SDT to guide treatment.

## 1. Introduction

Diabetes is one of the leading causes of sickness, decreased quality of life (QoL), and deaths worldwide [1]. It is also among the top 10 causes of death globally [2, 3]. Besides, diabetes is a chronic noncommunicable disease (NCD) that occurs when the pancreas produces insufficient insulin (the hormone that regulates the body's blood sugar level) or the body cannot effectively utilize the insulin produced [4]. Thus, diabetic patients usually take insulin daily to help absorb food nutrients into their system [5]. Globally, there were about 476 million diabetes cases, of which the incidence was about 22.9 million in 2017 [6]. Accordingly, over 1.37 million people died from the disease and its complications, with over 67.9 million disability-adjusted life years (DALYs) in 2017 [6]. The presence of diabetes exposes an individual to a 2-3-fold risk of all causes of death among adults [4]. Moreover, diabetes and cardiovascular diseases (CVD), respiratory diseases, and cancers account for over 80% of premature deaths from NCDs globally [7, 8]. Unfortunately, research shows that the condition will rise to over 693 million cases by 2045 if adequate measures, including efforts to ensure adequate self-management practices and medication adherence, are not implemented [9].

Patients with diabetes experience worse QoL than those without chronic diseases [10–12]. The QoL (physical and social functioning and perceived physical and mental well-being) of diabetic patients are mostly affected due to the management process and the need to adjust to diabetes management demands [7, 13, 14]. Thus, diabetic patients must consciously balance insulin intake and other management interventions [15]. Moreover, the psychosocial burden of living with diabetes usually affects the self-care behaviour, medication adherence, and QoL of diabetic patients [16]. The main target in diabetes care is maintaining blood glucose levels in a healthy range to avoid long-term diabetes complications through adhering to medications and appropriate self-management [17]. However, studies show low levels of medication adherence [18], poor self-management practices [19], and poor quality of life among diabetic patients [20]. Moreover, it seems there is a dearth of information on the most effective method of improving medication adherence, appropriate self-management practices, and QoL among diabetes patients [16].

Studies employed self-determination theory- (SDT-) based interventions to improve medication adherence, self-management practices, and QoL of diabetic patients [21–24]. The theory consists of three psychological needs, relatedness, competence, and autonomy, for optimal functioning [25]. Autonomy consists of diabetic patients' experiences and perceptions of control and self-initiation in line with their idea of self [26]. Additionally, competence develops from the need to be effective in diabetes management practices and the praise or appreciation of such excellence. Also, relatedness involves the need to “experience love and care as well as to express love and care towards others” [27].

Besides, the SDT improved the quality of life of diabetes patients in Norway [28]. Also, Raaijmakers et al. [29] found that self-determination regarding type 2 diabetes care contributed to improved QoL. Moreover, the SDT improved physical activities among diabetic patients in France [30] and Uganda [31]. Although studies have linked some constructs of the SDT and QoL of adults with diabetes, the effectiveness of SDT in improving the QoL and self-management practices of adult diabetic patients is not pronounced in the literature. This scoping review examined available evidence on the link between SDT and QoL of adults with diabetes.

## 2. Methods

This scoping review was conducted following the six-stage framework by Arksey and O'Malley [32]. The framework was adopted because it helps to assess the literature to examine what has been done and identify the gaps in knowledge that need attention [33]. Arksey and O'Malley [32] suggest that the following stages should be followed in conducting a scoping review: (1) identifying and stating the research questions; (2) identifying relevant studies; (3) study selection; (4) data collection; (5) data summary and synthesis of results; and (6) consultation.

In the first stage, we identified and drafted the research questions to guide the study: (1) How effective is SDT in improving diabetes self-management? (2) How effective is SDT in improving treatment adherence? and (3) How effective is SDT in improving the QoL of adult diabetic patients?

## 3. Identification and Selection of Studies

### 3.1. Search Strategy

Two authors (PO and HKK) conducted a preliminary literature search on the topic to set the inclusion and exclusion criteria. Furthermore, we expanded and refined our search strategy with expert help (an academic librarian at the University of Cape Coast). In addition, we conducted a vigorous literature review of published articles in four electronic databases (PubMed, JSTOR, Central, and ScienceDirect). We expanded the search via hand search to include other unpublished sources. The search strategy included literature from January 2011 to October 2021 using key search words and Boolean logic. Also, a free web-based search was conducted to retrieve other relevant materials. Also, Google Scholar and Z-library were searched for additional records. Furthermore, reference lists of eligible records were checked for other relevant articles.

The final search was completed on October 28, 2021. Titles and abstracts of studies retrieved were read, and only studies relevant to the study were considered. Six keywords were used in the search strategy: (“Self-determination Theory” OR “diabetes” OR “Application of Self-determination Theory” OR “QoL” OR “Diabetes self-management” OR “Diabetes medication adherence) AND (“Adults” OR “grownups” OR “people aged 18-75 years” OR “grown people”).

### 3.2. Eligibility Criteria

Studies were included if conducted among adult diabetic patients (type 1, type 2, and gestational diabetes), aged 18-75 years, measured at least one SDT-based motivational construct, and published online between January 2011 and October 2021 (with no limit concerning the start date). Also, the authors must have explicitly mentioned SDT as the framework for a study to be included.

### 3.3. Exclusion Criteria

We excluded studies that did not specify the study population and those that were not published in English. Additionally, nonprimary studies (systematic reviews and scoping reviews) and studies that used SDT-based measures but employed motivational interviewing as their guiding framework with no reference to SDT were excluded.

### 3.4. Procedure

**Figure 1:**
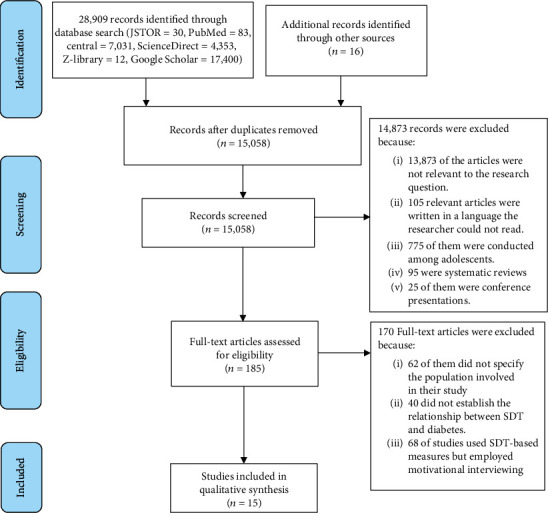
PRISMA flow diagram of records and screening process.

We used the eligibility criteria of the current study to scan the titles and appraise the abstracts of the identified literature for full-text review. We further scanned and manually screened the references of all included literature to add relevant studies to our review. Two of the current study's authors (PO and HKK) did the full-text review independently. The authors later met, reconciled the differences, and agreed on the included studies. We then developed a data extraction sheet with the following categories: author, year of publication, study title, country, population, study design, sample size, sampling strategy, and summary of findings (see Table 1 in the Appendix). Three of the study's authors (PO, HKK, and JOS) extracted the data independently. They later settled the differences to obtain a final result for the study. We involved third (EWA) and fourth (PYAA) reviewers to settle differences where there was disagreement in the findings of the three authors. One of the authors (PO) drafted the final extracted table (Results). All the authors read through the final draft results and ensured the findings reflected the agreed results. We finally carried out a thematic analysis and synthesis and presented the results. Additional consultations were made with subject experts to enhance the review. We used the PRISMA flow diagram to keep records and also screen the identified records (see Figure 1).

## 4. Results

The initial search in JSTOR, PubMed, Central, ScienceDirect, Google Scholar, Google, and Z-library produced 28,909 records (JSTOR = 30, PubMed = 83, Central = 7,031, ScienceDirect = 4,353, Z − library = 12, and Google Scholar = 17,400). Additional 16 records were identified through other sources. After removing duplicates using the Mendeley software, 15,058 records were available for screening. Furthermore, 185 pieces of literature qualified for full-text analysis for eligibility. We finally used 15 full-text studies in our thematic analysis and synthesis (Figure 1 details the screening process).

### 4.1. Characteristics of Included Studies

The included literature includes studies conducted among diabetic patients 18-75 years old using SDT to determine or improve QoL, diabetes self-management practices, and diabetes medication adherence. Out of the 15 included studies, five were experimental studies, one was a descriptive explorative qualitative study, five were cross-sectional studies, and one was a longitudinal study (Table 1 in the Appendix). Four studies addressed SDT and QoL among patients with diabetes, two explored SDT and treatment adherence, and 11 focused on SDT and diabetes self-management practices. Based on the research questions, three main themes were derived from the reviewed studies: (1) SDT and diabetes self-management; (2) SDT and diabetes treatment adherence; and (3) SDT and QoL among diabetic patients.

### 4.2. SDT and Diabetes Self-Management

Eleven [34] studies applied SDT to improving diabetes self-management. Lack of autonomy hindered diabetes self-management [35]. Also, autonomous motivation, perceived competence, and relatedness influenced the higher frequency of vigorous PA among people [31]. The application of SDT was effective in improving PA among diabetic patients in five studies ([11, 30, 36–38]). Besides, SDT was also influential in improving dietary self-care among diabetic patients [39] (see Table 1 [Appendix]).

### 4.3. SDT and Treatment Adherence

The adoption of an SDT-based intervention effectively motivated diabetic patients to comply with lifestyle recommendations and avoided noncompliance behaviours in one study [40]. Also, SDT-based intervention predicted that patients with less intrinsic motivation and a low relatedness score report a higher rate of nonadherence to diabetes treatment [41].

### 4.4. SDT and QoL of Patients with Diabetes

We summarized the findings on the effectiveness of SDT in determining or improving the QoL of diabetic patients. A study revealed that diabetic patients who did not receive autonomy support from their healthcare providers experienced anxiety about their health status and frustration with the care quality [35]. Also, patients who engaged in SDT interventions experienced new life possibilities and accepted their condition [28]. Further, diabetic patients who participated in the SDT intervention reported relatively dominant control motivation to comply with lifestyle recommendations and experienced improved health and QoL [40]. Also, diabetic patients who participated in the SDT intervention developed increased self-esteem. and vice versa [42] (see Table 1 in the Appendix).

## 5. Discussion

This scoping review determined the effectiveness of SDT in improving the QoL of diabetic patients. Our paper also explored the effectiveness of SDT in improving treatment adherence and appropriate self-management practices among diabetic patients. We highlighted several findings. Firstly, we found that a lack of autonomy support impedes diabetes self-management, whereas the autonomy support component of the SDT effectively improved PA among them. Secondly, SDT effectively increased appropriate dietary self-care practices among diabetic patients. Thirdly, SDT interventions effectively guided diabetic patients to develop the willingness, mastery, and connection to comply with prescribed medication and treatment methods. Fourthly, diabetic patients in SDT interventions developed the dominant controlled motivation to comply with their recommended medications. Lastly, SDT was effective in improving the QoL of diabetic patients.

### 5.1. SDT and Diabetes Self-Management

We found that a lack of autonomy support impedes diabetes self-management and that the autonomy support component of the SDT is more effective in improving PA and appropriate dietary self-care practices in diabetic patients. Our findings agree with studies conducted in South Africa [35] and China [34]. The diabetic patients in the South African study who received no adequate autonomy support from healthcare providers experienced more difficulty in effectively managing themselves than those who received autonomy support. However, there was an increase in diabetes self-management scores among autonomous support groups in the Chinese study. The similarities could be that when diabetes patients feel more autonomous (are willing to initiate an action without being forced to do so) in their management process, they tend to take control of their management process [38]. Typically, diabetes patients are challenged with the high cost of healthy foods, difficulty in giving up on unhealthy lifestyles, busy work schedules, side effects of medications, and accessibility of diabetic management services [43]. Perhaps autonomy support increases their willingness to continue appropriate management practices despite their challenges. The current findings imply that diabetic patients need a sense of desire to comply with diabetes self-management protocols to improve their health. However, contrary to the present results, Liu et al. [34] found autonomy support insufficient to promote appropriate self-management practices among diabetic patients, but other factors such as self-efficacy, knowledge, skill, family, and peer support.

Also, the current finding where autonomy supports improved PA is similar to that of other studies [31, 37, 38, 44] which reported higher PA among people with autonomy support and low PA among those with no or less autonomy support. Perhaps people who receive autonomy support engage in PAs willingly to satisfy their desires [45]. This finding may imply that diabetes self-management interventions that fail to provide autonomy support to their patients could experience a decrease in appropriate self-management practices among their participants.

Furthermore, we found that SDT (competence, relatedness, and autonomy) effectively increased appropriate dietary self-care practices among diabetic patients. Nouwen et al.'s [39] findings affirm those of the current study. They found diabetes patients in SDT intervention adopt healthy dietary practices. Perhaps SDT-guided diabetes management interventions promote patients' autonomy and help develop skills for healthy dietary patterns.

### 5.2. SDT and Medication Adherence

We found that SDT interventions effectively guided diabetic patients to develop willingness, mastery, and connection to comply with prescribed medication and treatment methods. Our findings are similar to those of other studies [40, 46]. This finding could be because SDT-based interventions equip diabetic patients to own their management interventions, feel supported, and help them develop competence in their management programs [44, 47]. However, Rajab et al. [41] found otherwise in their study. They found that patients with low autonomy and relatedness scores did not comply with their medication due to poor intrinsic motivation and relatedness [41]. Probably, when diabetic patients satisfy autonomy (free will), develop mastery (competence), and feel love and care from family and healthcare providers, they are more likely to comply with their medications and treatment routine that could improve their health and QoL. Also, contrary to our findings, Aloudah et al. [46] found higher treatment adherence among diabetic patients who observed others adhering to their treatment routines than those in SDT intervention. The divergent views call for a combination of therapies, such as SDT and imitation, to achieve treatment adherence and attendant health-improved outcomes in patients.

### 5.3. SDT and QoL among Diabetic Patients

We found that SDT effectively improves the QoL of diabetic patients. This finding could be because the theory improves patients' competencies, guides them to take voluntary actions, and makes them feel loved and supported [42]. Our finding implies diabetic patients could develop a high QoL if SDT guides diabetes management interventions. Our finding aligns with other studies [12, 35, 40, 42, 48]. These studies reported that diabetic patients in SDT interventions developed new life possibilities and exercised control over their new lives.

## 6. Limitations

This scoping review has provided insight into the effectiveness of SDT in improving QoL, diabetes treatment adherence, and diabetes self-management. However, there are a few limitations to the study. We included only open-access articles that were published in English. There is a possibility that we missed some vital literature.

## 7. Conclusion

The application of SDT can effectively improve appropriate self-management practices among diabetic patients. This implies that when interventions are implemented to enhance the autonomy, competence, and sense of connectedness (relatedness) among diabetic patients, they are more likely to adhere to the recommended diabetes self-management practices. This means that the economic burden on diabetes management, morbidity, and mortality cases associated with diabetes may be reduced globally. Furthermore, SDT was found to be effective in improving diabetes treatment adherence. Therefore, should SDT be used in daily diabetes care and interventions, more patients with diabetes may comply with diabetes medications and other treatment routines. We theorized that adopting SDT in the diabetes management process may improve patients' mental, social, and physical well-being and enable them to contribute effectively to society.

## Figures and Tables

**Table 1:** Effectiveness of SDT in diabetes self-management among adults.

Author	Year	Study title	Country	Population	Study design	Sample size	Sampling strategy	Summary findings
Murphy et al.	2015	A qualitative study of the experiences of care and motivation for effective self-management among diabetic and hypertensive patients attending public sector primary health care services in South Africa	South Africa	Hypertensive and diabetic patients 18 years and above.	Qualitative study comprising individual, in-depth interviews with hypertensive and diabetic patients	22	A criterion sampling procedure was used to select patients who had diabetes and/or hypertension	Patients experience multiple impediments to effective self-management of diabetes and behaviour change, including poor health literacy, a lack of self-efficacy, and perceived social support. Most patients reported not having received adequate information, counselling, or autonomy support from their healthcare providers. Their experiences suggest that the current approach to chronic care largely fails to meet patients' motivation needs, leaving many of them feeling anxious about their state of health and frustrated with the quality of their care.
De Man et al.	2020	What motivates people with (pre)diabetes to move? Testing SDT in rural Uganda	Uganda	Adults aged 30-75 years in two rural districts in eastern Uganda	The study was a cluster-randomized adaptive implementation trial	794	Cluster sampling technique	Their findings suggest that different types of motivation regulate different domains and intensities of PA. A higher frequency of vigorous PA—which was linked to a lower HbA1C and FPG—was predicted by autonomous motivation but not by controlled motivation. Perceived competence and perceived relatedness predicted autonomous motivation. Autonomous motivation functioned as a mediator between those needs and PA behaviour.
Karlsen et al.	2018	New possibilities in life with type 2 diabetes: experiences from participating in a guided self-determination program in general practice	Norway	Adults with type 2 diabetes mellitus	Descriptive and explorative qualitative approach	16	Purposive and convenient sampling technique	The findings indicate that the participants experienced new life possibilities after participating in the SDT program, which positively influenced their motivation for self-management. Through reflections on how to live with diabetes, the participants reinterpreted their life with diabetes by gradually developing a closer relationship with the disease and moving towards acceptance. Dialogue with nurses was seen to have helped support the participants to become more self-determined.
Liu et al.	2018	Effectiveness of self-management behaviour intervention on type 2 diabetes based on SDT	China	Adult diabetic patients in 3 selected communities in Beijing	Pretest posttest intervention study	3 communities	Convenient sampling method	The HbA1c and self-management scores increased higher in the autonomy support group than in the other two groups. After the intervention, the control rate in the autonomy support group was higher than in the other two groups. The value in the autonomy support group was higher than in the routine intervention group. Baseline self-management behaviour, self-efficacy, knowledge, skill, family support, autonomy support, peer support, and age were positively correlated with the change in behaviour.
Gourlan et al.	2016	Motivational profiles for physical activity practice in adults with type 2 diabetes: an SDT perspective	France	Adult type 2 diabetics patients in a diabetes care Centre in a French hospital	Cross-sectional design	350	—	Participants with “high combined” and “self-determined” profiles reported higher perceived competence and longer leisure-time PA practice in comparison to those with a “moderate” profile. This study highlights the necessity of adopting a person-centred approach better to understand motivation towards PA among type 2 diabetes patients.
Sebire et al.	2018	“I've made this my lifestyle now”: a prospective qualitative study of motivation for lifestyle change among people with newly diagnosed type two diabetes mellitus	England	Newly diagnosed adults with type 2 diabetes and were participants in the early ACTID trial	Randomized control trial	593	Purposive sampling	Applying SDT, it was found that many participants reported relatively dominant controlled motivation to comply with lifestyle recommendations, avoid their non-compliance being “found out” or suppress guilt following lapses in behaviour change attempts. Such narratives were accompanied by experiences of frustrating slow behaviour change progress. More autonomous motivation was expressed as something often achieved over time and reflected goals to improve health, QoL, or family time.
Rajab et al.	2020	Barriers to initiation of insulin therapy in poorly controlled type 2 diabetes based on self-determination theory	Iran	Patients with type 2 diabetes who had indications for insulin therapy	Descriptive cross-sectional study	151	Random sampling	The findings suggested that the patient's intrinsic motivation was less than their extrinsic motivation. It was observed that patients do not properly understand their illness due to the low score of relatedness representative of patients' and care providers' relationships.
Halvari et al.	2017	Physical activity and motivational predictors of changes in health behavior and health among DM2 and CAD patients	Norway	Adult patients with both type 2 diabetes and coronary artery disease (CAD)	Clinical trial	108	Purposive sampling	The data supported the SDT process model, in which the effect of the intervention significantly predicted indirect changes in behaviour and health through motivation variables. Considering the moderate to large effects on increases in motivation, behaviour, and health, promoting organized physical activity programs that are perceived as need-supportive may have important health implications for patients with DM2 and CAD.
Yun et al.	2020	Can autonomy support have an effect on type 2 diabetes glycemic control? Results of a cluster randomized controlled trial	China	Patients with type 2 diabetes	Cluster randomized controlled trial	364	Purposive and convenient sampling	Patients in an autonomous support group (ASP) achieved better HbA1c reduction at the end of intervention than those in the usual care group (UCG) and successfully maintained it for up to 6 months. However, patients in a social support group (SSG) did not experience a significant change in HbA1c at 3 or 6 months when compared with patients in UCG. Besides, patients in both the SSG and ASG experienced an improvement in exercise at 3 months. Patients in ASG sustained improvement in exercise for up to 6 months but those in the SSG did not. Autonomy support for patients with type 2 diabetes could help achieve glycaemic control at the end of the intervention and successfully maintain it for up to 6 months.
Nouwen et al.	2011	Longitudinal motivational predictors of dietary self-care and diabetes control in adults with newly diagnosed type 2 diabetes mellitus	Netherlands	People newly diagnosed with type 2 diabetes	Longitudinal study design	237	Purposive sampling	Dietary self-care was longitudinally associated with self-efficacy, self-evaluation, autonomy support, and autonomous motivation. The results indicate that autonomy support, self-efficacy, and self-evaluation are key targets for interventions to improve dietary self-care.
Koponen et al.	2018	Success in increasing physical activity (PA) among patients with type 2 diabetes: a self-determination theory perspective	Finland	Patients with type 2 diabetes	Cross-sectional survey	1256	—	The findings of this study supported SDT by showing that autonomous motivation was the strongest predictor of success in increasing PA among people with type 2 diabetes. The autonomous motivation was associated with success in increasing PA even after the effect of other important life-context factors was controlled for.
Koponen et al.	2017a	Determinants of physical activity among patients with type 2 diabetes: the role of perceived autonomy support, autonomous motivation, and self-care competence	Finland	Patients with type 2 diabetes	Cross-sectional survey	5167	Purposive sampling	Of all measured explanatory factors, autonomous motivation was most strongly associated with engagement in PA. Autonomous motivation mediated the effect of perceived autonomy support on patients' PA. Thus, perceived autonomy support was associated with the patient's PA through autonomous motivation. Interventions for improved diabetes care should concentrate on supporting patients' autonomous motivation for PA. Internalizing the importance of good self-care seems to give sufficient energy to maintain a physically active lifestyle.
Koponen et al.	2017b	Quality of primary health care and autonomous motivation for effective diabetes self-management among patients with type 2 diabetes	Finland	Patients with type 2 diabetes	Cross-sectional survey	2866	Purposive sampling	Autonomy support from one's physician was most strongly associated with autonomous motivation (self-regulation) for effective diabetes self-management among patients with type 2 diabetes.
Mohn et al.	2017	The effect of guided self-determination on self-management in persons with type 1 diabetes mellitus and HbA1c ≥64 mmol/Mol: a group-based randomized controlled trial	Western Norway	Adults (all Caucasian) aged 18–55 with type 1 DM for at least 1 year and HbA1c ≥64 mmol/Mol	Prospective randomize control trial	178	Random assignment	Participants in the guided self-determination group training (GSD-GT) group exhibited a significant reduction in diabetes-related distress relative to the control group (CG) the GSD-GT group showed an increase in self-esteem relative to the CG.
Güil Oumrait et al.	2020	Can self-determination explain dietary patterns among adults at risk of or with type 2 diabetes? A cross-sectional study in socioeconomically disadvantaged areas in Stockholm	Sweden	Adults at risk of and with type 2 diabetes from two socioeconomically disadvantaged Stockholm areas	Quantitative cross-sectional design	147	Purposive sampling	Two dietary patterns (healthy and unhealthy) were identified. The competence construct was most strongly associated with a healthy diet. Autonomous motivation and competence mediated the effect of relatedness on diet behaviour. In conclusion, social surroundings can promote adults at high risk of or with type 2 diabetes to sustain healthy diets by supporting their autonomous motivation and competence.

## Data Availability

All papers included in this work are available online and can be accessed based on their Open Access policies.
